# Childbirth violence-based negative health consequences: a qualitative study in Iranian women

**DOI:** 10.1186/s12884-021-03986-0

**Published:** 2021-08-19

**Authors:** Ziba Taghizadeh, Abbas Ebadi, Molouk Jaafarpour

**Affiliations:** 1 Nursing and Midwifery Care Research Center, Tehran University of Medical Sciences, Tehran, Iran; 2 Behavioral Sciences Research Center, Life Style Institute, Baqiyatallah University of Medical Sciences, Tehran, IR Iran; 3 Nursing Faculty, Baqiyatallah University of Medical Sciences, Tehran, IR Iran; 4 Department of Reproductive Health, Nursing and Midwifery Faculty, Tehran University of Medical Sciences, Tehran, Iran

**Keywords:** Childbirth, Women, Violence, Abuse, Consequence, Iran, Qualitative research

## Abstract

**Background:**

Violation of mothers' rights during childbirth is a global problem that often silently torments women in many parts of the world. The aim of this study was to explore negative health consequences due to childbirth violence based on mothers' perceptions and experiences.

**Methods:**

To achieve rich data, an exploratory qualitative study was carried out in 2019 on 26 women with childbirth violence experience who had given birth in hospitals of Ilam, Iran. Data were collected using semi‑structure in‑depth interviews (IDIs) and a purposive sampling. Participants were asked about their experiences and perceptions of negative health consequences due to childbirth violence. Data were analyzed by conventional content analysis based on Graneheim and Lundman approach. MAXQDA (v.18) software was used for better data management.

**Results:**

Final codes were classified into 9 sub-categories and 3 main categories including maternal and newborn injuries, weakening of family ties, sense of distrust and hatred. These findings emerged the theme: negative health consequences.

**Conclusions:**

This study broke the silence of abused mothers during childbirth and expressed the perspective of mothers who suffered childbirth violence as a routine phenomenon in maternal care, and a serious threat to the health of mothers, newborns and families. Findings of this study can be a warning for maternity health system, monitoring and support structures as well as health policy-makers to seriously plan to prevent and eliminate this problem.

## Background

Although overt violence has always been the focus of research, recently many researchers have shown interest in childbirth violence. Many women experience childbirth violence during labor and birth silently in the following forms: being uncared for, and even being hurt [1–7]. Childbirth violence is an obvious violation of women's fundamental rights and their independence which poses serious threats to mothers and their newborns that in a sensitive and vulnerable period [8–10].

Women’s experiences of mistreatment during facility-based delivery can be expressed as physical, verbal abuse, sexual abuse, stigma, discrimination, failure to meet standards of care and attention – such as neglect and abandonment, lack of informed consent and confidentiality, privacy, and confidentiality violation, medical procedures conducted without consent– the poor rapport between woman and healthcare providers and the conditions and constraints of health systems [11]. Many researchers believe that childbirth violence is a manifestation of structural violence, especially structural gender inequalities in society that frame maternity care practice in health systems, proving that women's lives are not valued by larger social, economic, and political structures. [10, 12–14].

Numerous studies worldwide emphasize that women often experience disrespect and abuse during childbirth [15–23]. Studies report that 20%, 20%–28%, 78%, and 98% of women in Kenya, Tanzania, Ethiopia, and Nigeria experience childbirth violence [15, 24–26]. A new WHO-led and multi-country study has demonstrated that more than one-third of women experienced mistreatment during childbirth in health facilities [27].

Although Iran is a successful country in reducing maternal mortality and achieving MDG5 with a 75% reduction in MMR by 2015 [28, 29], the quality of care and women's childbirth experience has been less emphasized. Several studies in Iran indicate poor quality of maternal care [30, 31]. Lack of women-centered care, focus on medical interventions and birth without the presence of a spouse or favorite companion are among the problems of maternal care. Based on a new study among a group of Iranian women, 75% of women reported perceived disrespectful maternity care, such as not being allowed to choose labor positions and move during labor [32]. In addition, failure to informed decision-making about the delivery method [33], low freedom of choice and decision-making in access to health services [34, 35], and the lack of a complaint management system have been reported in Iranian health settings [35].

Mistreatment of women during childbirth is in stark contrast to the moments of love and joy of giving birth to a newborn and can physically harm women and babies and lead to emotional and psychological trauma to women. Women who are restricted to certain positions suffer from back, hip, and pelvic injuries due to pressure in an unnatural position [36]. Raj et al. (2017) stated that mistreatment of women is associated with increased risk for maternal health complications, such as obstructed labor and postpartum hemorrhage [37]. Some studies show that negative experiences of women in obstetric care can lead to postpartum psychological problems, including postpartum depression, [38] post-traumatic stress disorder, deep feelings of guilt and sadness, and feelings of helplessness. In the long-term, the desire for pregnancy decreases, while the tendency to cesarean section, negative feelings, thoughts about the baby, and breastfeeding problems increase [39–41].

Also, childbirth violence reduces mothers' satisfaction and trust in the health systems [42], potentially deterring women from seeking medical care in the future, leading to severe negative health consequences. Bohren et al. (2014) found that disrespectful and abusive care is a major barrier to skillful delivery, reporting that health workers as impolite, irascible, critical with a negative attitude, and generally useless [43].

However, in recent years there has been a progressive increase in the volume of studies on reporting prevalence and determinants of childbirth violence worldwide, although few researchers have evaluated the consequences of childbirth violence on mothers and infants [6]. Since a qualitative study explores what lies behind women's experiences of violence [44] and given that few global qualitative studies have assessed the various dimensions of the consequences of childbirth violence, this paper aimed to explore the negative health consequences of childbirth violence based on the experiences of women. The results of this study can be an effective step in attracting the attention of implementers and policymakers to plan and find solutions for preventing childbirth violence.

## Methods

### Study design

An exploratory qualitative study was employed among women who had given birth in one of the hospitals in Ilam city, Iran.

### Setting and participants

Our study was conducted in Ilam City, the center of Ilam Province, in west of Iran with an area of 20.133 km2 and a population of 235,144 people. Ilam has four maternity hospitals, including public educational hospital of Taleghani (referral maternity hospital in the province), semi-public hospital of Zagros and two private hospitals of Kowsar and Ghaem. Participants were recruited from those who had given birth in one of the hospitals in Ilam. The women were selected using purposive sampling based on the maximum variation method. Inclusion criteria were women of reproductive age (18–49) with previous experience of delivery (vaginal delivery or emergency cesarean section) in the hospital, experience of childbirth violence (based on self-reporting), and speaking in Persian. They were able to talk and provide us rich information. In order to avoid recall bias, postpartum women were included from one week to 3 years after delivery.

### Data collection

This study used a qualitative approach for data collection using semi‑structured in‑depth interviews (IDIs) with women of reproductive age from August to December 2019. In the qualitative studies, sampling is purposeful and the researcher is looking for participants who have rich experiences about the desired phenomenon. Given the cultural aspect of childbirth violence and the absence of a tool to measure this phenomenon, the study managed to identify women subjected to childbirth violence using only a few general questions and mothers' self-reporting. Therefore, women were asked general questions such as “Were you annoyed since you were admitted to the hospital, during giving birth until you were discharged from the hospital”? or “Did certain behaviors of the caregiver make you angry?” In this regard, women who had unpleasant and annoying experiences were invited to the interview and asked to explain more about their unpleasant experiences. The negative health consequences of the childbirth violence theme emerged in the earliest interviews; however, it was explored more explicitly and carefully in the next interviews. The next questions were asked based on the participants' responses to the first question. The interviews were conducted by the corresponding author (M.J), who is a faculty member and a Ph.D. student in reproductive health with adequate experience in qualitative research. All stages of data collection and analysis were supervised by the first author of this article (Z.T) as a faculty member, a Ph.D. in reproductive health and an experienced researcher in qualitative studies.

With the consent of the participants, the interviews were conducted in private rooms at the health centers of Ilam, Iran, to which women were referred for postpartum care, the participants' home, or any place the participants felt comfortable. Given that the researcher was from Ilam, her experience of childbirth in these hospitals, the relative childbirth violence she had undergone, and her expertise in reproductive health to assist mothers with questions about childbirth, she managed to communicate deeply and intimately with the mothers in the study, gain their trust, and extract their experiences and feelings in an intimate atmosphere during the interviews. In-depth interviews lasted approximately 30–90 min and were all recorded. Sampling ended when no new data was available and data saturation was obtained.

### Data analysis

In this study, a qualitative conventional content analysis approach was used, as described by Graneheim and Lundman (2004). The analysis process was performed simultaneously with data collection. At the end of each interview, all audio files and notes of the interviews were handwritten and typed verbatim. The typed texts were then read several times to overview their contents. The semantic units and the initial codes were extracted from the texts based on the inductive method. The initial codes were categorized in subcategories and categories based on similarities and differences. Finally, the basic categories formed the main theme [45]. After typing the transcripts of interviews, MAXQDA v.18 software was used for better data management.

### Data trustworthiness

Qualitative research will be valid when it accurately embodies and expresses the experiences of study participants. Polite (2017) proposed five criteria to improve the degree of trustworthiness of qualitative research, including credibility, dependability, transferability, conformability, and authenticity. The member check was conducted to increase the credibility of the data. Therefore, after the analysis, the results were referred to several participants to check if the findings are the same as what they intended when expressing their experiences. Also, the codes were independently reviewed by other experts and the research team.

In order to ensure data dependability, all interviews were carefully recorded and written, and during the research report, the participants’ conversations were quoted. Also, the researcher provided the research results to an external supervisor to audit the research.

To increase the transferability of the study, the researcher tried to provide a detailed description of the research path and writing the dissertation to enable other researchers to follow the research steps. Therefore, the researcher tried to increase the transferability of the data by a full description of the participant’s characteristics, the sampling method, and the place of sampling. Also, the samples were selected with a maximum variation of age, education, economic status, and place of delivery.

The clarity of the research method and all stages of the work allowing other experts to follow and judge increased the confirmability of the study. Also, reviewing the transcripts of the interviews and the extracted codes by the external observer improved the findings.

The researchers promoted research authenticity by appropriate selection of participants and prolonged engagement with them throughout the study, as well as reviewing the extracted codes and categories by the participants and members of the research team.

## Results

Data were extracted from the analysis of interviews with 26 participants. Table1 presents the sociodemographic characteristics of participants. Nine subcategories and three main categories, including maternal and newborn injuries, weakening of family ties, sense of distrust, and hatred, were extracted from the textural analysis of the interviews on the consequences of childbirth violence. These findings constituted the theme of negative health consequences (Table 2).

**Table. 1:** Sociodemographic characteristics of participants

Variable	Frequency (%)
**Age range (years)**	
20–24	5 (19.2)
25–29	6(23)
30–34	7(26.9)
35–39	4(15.4)
40 +	4(15.4)
**Parity**	
The first birth	16(61.5)
The second birth	6(23)
The third birth or more	4(15.4)
**Type of delivery**	
Vaginal delivery	16(61.5)
Cesarean section	10(38.5)
**Place of delivery**	
Public hospital	18(69.2)
Semi-public hospital	2(7.7)
Private hospital	6(23)
**Educational level**	
No formal education	2(7.7)
High school diploma or less	11(42.3)
Higher education	13(50)
**Occupational status**	
Housewife	16(61.5)
Employee	10(38.5)
**Economic status**	
Favorable	7(26.9)
Average	13(50)
Unfavorable	6(23)

**Table. 2:** Subcategories, categories and theme of negative health consequences due to childbirth violence based on mothers' perceptions and experiences

Sub- categories	Categories	Theme
Mother or newborn physical injuries	Maternal and newborn injuries	Negative health consequences
Psychological trauma for the mother		
Disrupting the attachment between mother and newborn	Weakening of family ties	
Disrupting the emotional- sexual relationship between woman and her spouse		
Distrust in health system	Sense of distrust and hatred	
Hatred of hospital and healthcare providers		
Negative reactions against healthcare providers		
Distrust in supportive structures		
Hatred of childbearing		

### Maternal and newborn injuries

This category contained two subcategories. These subcategories involved the physical injuries of the mother or newborn and psychological trauma for the mother.

According to the women's expression, serious injuries such as severe bleeding and vaginal hematoma due to healthcare providers' negligence have put women's health at risk during childbirth.

*“… There was not a drop of blood left in my body. I had lost so much blood and I didn’t know it! They said a vein had been torn. A vein in the birth canal had been torn and they realized it too late, despite telling them a lot, I am in severe pain… I was hospitalized for four days after that.” (Participant No.11, 24 years old).*

Also, fetal distress, increased cesarean section, rupture of the birth canal, following induction, and physical pressure on the abdomen during childbirth have been other serious risks that have threatened the lives of mothers.

*“For no reason, they augmented my labor pains by medication so that I could give birth earlier. But finally, I had a cesarean section because my baby was about to die.” (Participant No. 8, 27 years old).*

*“During childbirth, they pressed my abdomen hard, forcing the baby to be born. After the delivery, I started bleeding profusely … I had a severe rupture, so I had a lot of sutures. I was very disturbed after giving birth. For a few days, I couldn’t even sit.” (Participant No. 26, 29 years old).*

Some mothers experienced a variety of psychological trauma as a result of enduring childbirth violence. These include nightmares, panic, and fear of childbirth.

*“… I had a terrible nightmare of that night (delivery) for a few months.” (Participant No. 25, 31 years old)*

*“… When the word childbirth is brought up, I get goose bumps, all those scenes come before my eyes again and my whole body trembles. I still have nightmares.” (Participant No. 18, 23 years old)*

Severe stress, suicidal ideation, or running away from the hospital are other consequences of the violence of the care health providers described by the mothers.

*“Believe me, that night, if I could find a way, I’d escape! I even looked out the window, I said if there was a way, I’d even throw myself out of the window! I was so annoyed that night… They did not pay attention to me at all.” (Participant No. 2, 26 years old).*

One participant stated that she had witnessed a woman escape from the maternity ward due to a healthcare provider’s mistreatment.

*“There was a woman next to me. They shouted at her. She was very agitated and terrified. the door was open and she ran away.” (Participant No. 15, 30 years old)*

Threatening the mother to force her to carry out care orders by the healthcare providers has been accompanied by serious psychological trauma. Severe stress, panic, feelings of hopelessness, demoralization, and inability have been painful experiences reported by some mothers, associated with threatening and intimidating mothers with issues, such as asphyxia and fetal death or abandonment of the mother if she does not cooperate with healthcare providers.

*“They told me that “if you don’t cooperate, if you don’t try, your baby will die and you will no longer hear the sound of his heart!” It was as if the world collapsed on my head! They said that and I just cried. I was feeling completely frustrated! It might be normal for them to say this, but it was very annoying for me, it was horrible, horrible.” (Participant No. 6, 29 years old).*

*“They agitated me a lot. Their talks got on my nerves, like a drill and it greatly demoralized me.” (Participant No.7, 29 years old)*

Many mothers complain about feelings of intense homesickness and loneliness due to poor hospital delivery conditions.

*“I just shed tears of loneliness. I just looked at the door and the walls, felt blue and cried. Giving birth in such a place is damn horrible.” (Participant No.20, 30 years old)*

*“That environment and they were all strangers and I could not tell them everything that I needed. I was very lonely! The feeling of loneliness is so terrible!” (Participant No. 26, 29 years old).*

*Some participants stated that they felt humiliated and worthless due to being mistreated.*

*“That is, they treated me in such a way that I felt I was of no value to them. It was as if I were a non-living thing …Now, actually, the memory of that time still makes me feel somehow bad about myself.” (Participant No.4, 24 years old).*

### Weakening of family ties

Another serious consequence of childbirth violence is the weakening of family ties that consisted of two subcategories: Disrupting the attachment between mother and newborn and disrupting the emotional-sexual relationship between a woman and her spouse.

Impaired mother-infant emotional connection and poor breastfeeding are among the negative consequences of childbirth violence that mothers reported.

*“…I wasn’t with my baby for 4 days. My baby was hospitalized in the neonatal intensive care unit and I was in another ward. I don’t know what they fed him with.” (Participant No. 12, 28 years old).*

Another participant stated that: *“I suffered the worst during childbirth, now, when I look at my child and I remember that time, I feel very bad. Somehow I don’t enjoy raising him.” (Participant No. 9, 39 years old).*

The statements of some mothers in this study indicated that childbirth violence was associated with a disorder in the emotional-sexual relationship between the woman and her husband.

*“I hate having sex with my husband now (Participant No.3, 35 years old).), because I feel that if we hadn’t had this relationship, I wouldn’t have suffered so much and I would really say that I wish I hadn’t been married at all.” (Participant No.18, 23 years old).*

### Sense of distrust and hatred

According to women’s perceptions, a sense of distrust and hatred was an important consequence of childbirth violence that can affect women's health-seeking behaviors in the future. Thus, it indirectly endangers the health of women and their newborns. The subcategories of this category were eroding distrust in the health system and distrust in the supportive structures.

Childbirth violence has eroded mothers' trust in the hospital environment and healthcare providers; some even prefer home to a hospital for childbirth.

*“I no longer trust them. I know they are no different from each other. They are all just as cruel. If I want to have another child, I won’t go to the hospital at all. It’s much better at home.” (Participant No.14, 36 years old).*

One mother describes her experience with the hospital and her severe distrust in the health system: *“Now, I really tell all the family and acquaintances not to go to the hospital for childbirth. Because they treat you the worst there. A hospital is like quarantine, it is like a prison, and women are treated like criminals.” (Participant No.18, 23 years old).*

Hateful experiences and feelings of healthcare providers and the hospital are obvious in the participants' statements.

*“I hate them so much that I don't even want to pass through the hospital just a moment! I will never forgive them. They treated me very badly.” (Participant No. 24, 42 years old).*

Several participants stated negative reactions such as aggressive behaviors against the healthcare providers.

*“I had a big fight with a healthcare provider and even told her that I’d sue her. She was very rude and harsh. They should be treated just like themselves.” (Participant No. 13, 32 years old).*

*“They ignored me and my companions so much that my husband flied off the handle. He shouted at them and punched the door and the wall.” (Participant No. 23, 34 years old).*

Another experience of the participants in this study was the women's distrust in supportive laws, causing them to be frustrated with the violation of their rights.

*“All this says that you should respect the pregnant woman, treat her with respect and dignity, but where is it??! It is all words!” (Participant No. 2, 26 years old).*

Mistreatment of women during childbirth has led women to distrust supervisory and grievance redress structures.

*“My husband wanted to sue them, I told him it's no use. In the end, just we get tired. Because no one defends pregnant women and so, they treat you however they like.” (Participant No.14, 36 years old).*

*“No one supports us. You know that so many women are abused in the hospital during childbirth, but which law has addressed their problem?” (Participant No.26, 29 years old).*

Based on most participant’s experiences, childbirth violence turns the moments of love and joy of the baby's birth into the most tragic event in the mother's life, making women hate childbearing.

*“My delivery was the worst memory! The most tragic event! Giving birth in the hospital was the most horrible event possible for me. I hate any childbirth. I have repented to give birth again.” (Participant No. 18, 23 years old).*

*“They brought me a disaster that I say God forbid I give birth again. Just now I wish I would never have another baby!” (Participant No. 5, 23 years old).*

## Discussion

This qualitative study was conducted to explore the consequences of childbirth violence based on women's experiences. According to the results, the most important consequences of childbirth violence were maternal and newborn health injuries, weakening family ties, and forming a sense of distrust and hatred. All these negative consequences ultimately threaten the health of the mother and family directly or indirectly.

The findings of the current study expressed that childbirth violence can result in physical harm to women and babies, as well as psychological harm to women. Some participants reported experiencing excessive bleeding and vaginal hematoma due to negligent acts and abandonment by healthcare providers. Other women suffered injuries during labor and delivery, such as birth canal and perineum laceration, increased risk of cesarean section, and fetal distress due to unnecessary and risky interventions. Miller et al. (2016) state that two extreme situations on the continuum of maternal health care, including too little, too late, and too much, too soon, can directly contribute to poor outcomes. In other words, sometimes, women either suffered from inadequate resources with too late care or over-medicalization of normal childbirth, including excessive and inappropriate use of uncomfortable interventions. Both of these extremes can play a major role in increased maternal and neonatal morbidity and mortality. Healthcare providers’ neglect or abandonment can prevent timely or proper diagnosis and treatment of complications. Also, overuse procedures, including induction, augmentation, continuous electronic fetal monitoring, episiotomies, cesarean section, and enemas, can cause maternal or neonatal complications, such as fetal distress, uterine rupture, or perineal laceration [46]. Consistent with our finding, Raj et al. (2017) showed that women reporting mistreatment during childbirth were more likely to experience complications such as obstructed labor and excessive postpartum hemorrhage [47]. Therefore, the right amount of care should be provided at the right time and in a way that respects, supports, and promotes the mother's rights[46].

In addition to physical injuries, some participants experienced psychological harm as a result of childbirth violence. Some participants had experienced poor emotional conditions during hospitalization, such as feelings of intense homesickness and loneliness. Also, severe stress, nightmares, panic, fear of childbirth, demoralization, and inability were other consequences of childbirth violence in women. Mental health professionals have increasingly recognized birth trauma and negative birth experiences as factors that impair postpartum well-being that may require counseling or other treatments [36]. Silveira et al. (2019) showed that disrespect and abuse during childbirth increased the odds of postpartum depression [38]. Also consistent with our finding, Ayers et al. (2016) found that negative subjective birth experiences and an operative birth were strongly associated with the occurrence of post-traumatic stress disorder in the postnatal period [48] Moreover, the statements of one of the participants indicated that the feeling of worthlessness and humiliation induced by healthcare providers’ abuse during childbirth torments mothers for several years after delivery. Forssen et al. (2012) also achieved similar results that violating women's dignity during childbirth can lead to disempowering experiences for the rest of their lives [39].

According to this study, childbirth violence threatens women’s health and undermines family ties. The lasting emotional injuries due to violence inflicted on women during childbirth disrupts the bonding and the long-term attachment between mother and baby [36] as well as the emotional-sexual relationship between a woman and her husband [49]. Some mothers are so damaged that even the mother-baby bond, skin-to-skin contact and breastfeeding are affected. Some participants stated that recalling the painful memories of childbirth makes them feel annoyed with the baby and husband. Other studies support our findings [41, 50].

In addition, violent maternity care has led to women's distrust in healthcare system and the current supportive structures. As such, some participants described the hospital and healthcare providers as a hateful environment. Several studies stress the maternal distrust in the healthcare system due to childbirth violence as a powerful dissuasive, negatively affecting the future health-seeking behaviors in mothers, leading them to prefer home delivery [42, 51–53], where they can deliver in a preferred position, can cry out without fear of punishment, receive no surgical intervention, without any physical restraints [54]. The women's distrust in the healthcare system became even more apparent when some participants described giving birth at the hospital as the bitterest memory and event of their lives, while the birth of a baby could be the happiest and most enjoyable moments for them [36].

Furthermore, some participants stated there was no effective and accountable law to protect them against childbirth violence. They believed that the rules for honoring pregnant women are merely words, and in practice, the law is silent and does not support the rights of pregnant women. Distrust in regulatory and protection laws has caused mothers to be frustrated with the violation of their rights. Moore et al. (2011) believed that policies to protect mothers' rights are hardly enforced, and healthcare providers are scarcely held accountable for their disrespectful and abusive treatment of women during childbirth [55]. Kukura (2018) criticized the existing legal and regulatory structures, considering them insufficient and ineffective in handling cases of mistreatment of women. Thus, when women complain to legal authorities, they often face failure and non-fulfillment of their rights due to legal restrictions to compensate for the harms of childbirth violence, including inadequate access to representation in childbirth violence litigations, difficulty in establishing a cognizable claim, and difficulty in proving harm. Therefore, more advocacy is needed to hold legal authorities accountable for complaints and allegations of mistreatment of women during childbirth [36]. Similarly, Ishola et al. (2017) [53] noticed the poor performance of laws and policies in protecting mothers' rights, reporting results in line with the findings in this paper. Based on our study, distrust in the healthcare system and women's despair of protective laws made some women hate childbearing and decide to never have another birth that repeats negative experiences because they believed they could not change the existing situation given their perceived lack of voice. Shorey et al. (2018) found that a negative birth experience is related to the decision not to have another child, which agrees with our finding [56]. Similarly, other studies showed that unpleasant experiences of childbirth can negatively affect the decision about the next pregnancy [41, 57, 58].

Given the different conditions in health structures and hospital constraints and the contextual nature of childbirth violence, and the complex relationships between healthcare providers and maternal experiences, further exploratory studies on childbirth violence and its consequences are needed nationwide and worldwide.

Considering the serious consequences of childbirth violence, health executives need to put various policies on their agenda to protect women from childbirth violence. In order to timely and correct care and prevent unnecessary and harmful interventions, healthcare systems need to monitor the development and proper implementation of care guidelines more carefully. Clinical guidelines for maternity care should focus on less unnecessary interventions, respect for women's preferences, more mobility of low-risk mothers in labor, providing evidence-based maternity practices, and woman-centered care.

Also, considering that the attentive and respectful attitudes and behaviors of healthcare providers are very important in helping mothers have a sense of trust in the hospital environment, to whom everyone is a stranger and unfamiliar, healthcare systems should provide training for promoting communication and social skills and respect for the human dignity of the mother in stressful situations. This crucial issue has also been addressed in the study of Theuring et al. (2018)[59].

Health implementers should give continuous effort for addressing and eliminating health system constraints serious consideration since these restrictions, such as staff shortages, poor infrastructure, or lack of medications, in addition to being directly experienced by women in the form of mistreatment, can indirectly by creating a stressful work environment increase poor healthcare providers’ behaviors or even abuse of women. It should be noted that healthcare providers’ mistreatment of women is not necessarily intentional and may be accompanied by compassionate and respectful care. [60]. Furthermore, the development of the physical space of hospitals and the option of allowing one person to accompany women and emotionally supporting them during childbirth are other necessities that healthcare service implementers need to address appropriately. Childbirth in the presence of a favorite companion for several generations is practiced in European countries and is essential for creating an attractive and pleasant environment for mothers [16, 59, 61].

Finally, regulatory and supervisory laws must be enforced properly to continuously check the healthcare providers’ actions regarding respectful maternity care and how childbirth is handled. Also, grievance redress mechanisms following any mistreatment and ensuring professional standards of clinical care need to be firmly enforced [62].

## Conclusion

This study broke the silence of abused mothers during childbirth, and expressed the perspective of mothers about the physical and psychological injuries inflicted on mothers and newborns, and its consequences, such as the loss of maternal communications and distrust in and hatred of the maternity care system, support structures and finally hatred of childbearing. It is hoped that healthcare providers as well as policymakers will pay attention to appropriate and timely planning so that we no longer witness the bitter experiences of mothers and their families regarding childbirth**.**

### Strengths and limitations

To our knowledge, this study was conducted in Iran for the first time to explore experiences of women with consequences of childbirth violence. The need for this research was greatly felt because childbirth violence is commonly seen in the maternal care system in low and middle-income countries [63] without seriously addressing its consequences for mothers and newborns, just as mothers experience it. The findings of the study can be a serious warning for our healthcare system, monitoring and support structures and health policy-makers in this field that seriously plan to prevent and eliminate this problem.

Another strength of this study is that our participants were selected from multi-central maternity care (public, semipublic and private) and included both vaginal delivery and cesarean section.

Although we tried to observe maximum diversity in sampling, the non-generalizability and dependence of research results on conditions, especially place, were among the limitations of this study. The findings of the study, considering this limitation, may be useful for other researchers.

## Data Availability

The datasets used and analyzed during the current study are available from the corresponding author on reasonable request.
